# Posterior Teeth Occlusion Associated with Cognitive Function in Nursing Home Older Residents: A Cross-Sectional Observational Study

**DOI:** 10.1371/journal.pone.0141737

**Published:** 2015-10-29

**Authors:** Kenji Takeuchi, Maya Izumi, Michiko Furuta, Toru Takeshita, Yukie Shibata, Shinya Kageyama, Seijun Ganaha, Yoshihisa Yamashita

**Affiliations:** 1 Section of Preventive and Public Health Dentistry, Division of Oral Health, Growth and Development, Faculty of Dental Science, Kyushu University, Fukuoka, Fukuoka, Japan; 2 Kizuna-kai, Aso-kizuna Dental Clinic, Aso, Kumamoto, Japan; Taipei Veterans General Hospital, TAIWAN

## Abstract

Early detection and subsequent reduction of modifiable risk factors for cognitive decline is important for extending healthy life expectancy in the currently aging society. Although a recent increase in studies on the state or number of the teeth and cognitive function, few studies have focused on the association between posterior teeth occlusion necessary to maintain chewing function and cognitive function among older adults. This study examined the association between posterior teeth occlusion and cognitive function in nursing home older residents. In this cross-sectional study, 279 residents aged ≥60 years from eight nursing homes in Aso City, Japan participated in cognitive function and dental status assessments and completed a comprehensive questionnaire survey in 2014. Cognitive function was measured using a Mini-Mental State Examination (MMSE). Posterior teeth occlusion was assessed using a total number of functional tooth units (total-FTUs), depending on the number and location of the remaining natural and artificial teeth on implant-supported, fixed, and removable prostheses. Linear regression models were used to assess univariate and multivariate associations between total-FTUs and MMSE scores. Models were sequentially adjusted for demographic characteristics, number of natural teeth, socioeconomic status, health behaviors, comorbidities, physical function, and nutritional status. Among the 200 residents included in our analysis, mean MMSE scores and total-FTUs were 11.0 ± 8.6 and 9.3 ± 4.6, respectively. Higher total-FTUs were significantly associated with higher MMSE scores after adjustment for demographics and teeth number (B = 0.48, 95% confidence interval [CI] = 0.22–0.74). The association remained significant even after adjustment for all covariates (B = 0.25, 95% CI = 0.01–0.49). The current findings demonstrated that loss of posterior teeth occlusion was independently associated with cognitive decline in nursing home older residents in Japan. Maintenance and restoration of posterior teeth occlusion may be a preventive factor against cognitive decline in aged populations.

## Introduction

Cognitive impairment and dementia resulting from age-related decline in cognitive function are substantial public health concerns because they are common in older people and major causes of morbidity and mortality worldwide [1, 2]. Few treatments and health service settings have been effective for lower cognitive function levels [3, 4]. Therefore, early detection and subsequent reduction of modifiable risk factors for cognitive decline is important for extending healthy life expectancy in the currently aging society.

Growing evidence suggests that dental health may be an important factor associated with cognitive function in aged populations. Prospective studies in several countries have reported positive relationships between natural teeth number and cognitive function [5, 6]. In a prospective study of older Japanese adults, those with few remaining natural teeth and without dentures had a significantly higher risk of dementia onset than those with ≥20 teeth [7]. In particular, complete tooth loss may be an early marker of cognitive decline [8, 9]. Further, periodontal disease and caries, major reasons for tooth loss, are also reported to be related to cognitive decline in a prospective study of U.S. men [10].

Despite a recent increase in studies on the state or number of the teeth and cognitive function, few studies have focused on the association between posterior teeth occlusion necessary to maintain chewing function and cognitive function among older adults. In a Japanese rural community population study, an association between the pattern of posterior occluding pairs of natural teeth and cognitive function was tested, but a significant association was not found [11]. However, there was no reliable evidence from this study because of no consideration of the occluding pairs of artificial teeth and the number of natural teeth.

The aim of our study was therefore to examine the association between posterior occlusion with natural or artificial teeth and cognitive function in a nursing home population, including assessment of the number of natural teeth. We hypothesized that posterior occlusion with natural or artificial teeth would be more important for the individual’s cognitive function than the total number of natural teeth.

## Methods

### Study population

This cross-sectional study was conducted in eight nursing homes in Aso City, Kumamoto Prefecture, Japan, from February 2014 to June 2014. Nursing home residents aged ≥60 years capable of oral nutrient intake were enrolled along with their surrogate decision-makers. Residents using tube feedings or being cared for at a hospital or nursing home were excluded. In all, 279 residents from eight nursing homes participated in cognitive function and dental status assessments and completed a comprehensive questionnaire survey for the study. After excluding respondents with missing responses to questions on cognitive function (n = 19) and other covariates used in the analysis (n = 60), 200 respondents were included in the analysis. The study was conducted with the approval of the Kyushu University Institutional Review Board for Clinical Research. A written informed consent was obtained from all of the respondents. Only when respondents were unable or difficult to understand our explanation of the study, the written informed consent was obtained from surrogates or legal representatives.

### Cognitive function assessment

Trained nursing home care staff assessed cognitive function using the Mini-Mental State Examination (MMSE) [12], which included the domains of registration, orientation, attention and concentration, memory, language, and ability to follow simple commands. The score ranged 0–30 (higher scores represented better cognition). We used the MMSE score as a primary outcome measure.

### Dental status assessment

Dental status was clinically examined by one trained dentist. Posterior teeth occlusion was assessed using a total number of functional tooth units (total-FTUs), which were defined as the number of pairs of opposing natural teeth and artificial teeth on implant-supported, fixed (bridge pontics), and/or removable prostheses in posterior teeth occlusion [13]. The score ranged 0–12 (higher scores represented better occlusion). Therefore, a person with complete dentition scores of a perfect 12 (except for the third molars). We used total-FTUs as a primary predictor for cognitive function. The dentist also evaluated the number of remaining natural teeth and dentures use for each respondent during the dental examination. Based on the number of natural teeth, we divided the respondents into an edentulous group and groups with 1–9, 10–19, and ≥20 teeth.

### Covariates

We included established or suspected factors associated with dental status and cognitive function based on prior literature. Information on demographic characteristics, socioeconomic status, health behaviors, comorbidities, physical function, and nutritional status was obtained from a standardized questionnaire interview with respondents, or surrogates and caregivers by nursing home care staff and from information in nursing home medical records. Age (categorized as ≤84, 85–89, 90–94, or ≥95 years) and sex were used as demographic characteristics. Longest job, defined as the job being done for the longest time [14], was used to stratify participants into socioeconomic statuses: white-collar workers, blue-collar workers, and all others including the unemployed. White-collar workers included managers, professionals, office workers, and service and sales workers; blue-collar workers included skilled and elementary workers and agricultural, forestry, and fishery workers. Smoking and drinking history were recorded as health behaviors, and responses were dichotomized into ever or current smoker/never smoker and ever or current drinker/ never drinker. Information on physician-diagnosed cerebrovascular disease and other diseases comprising the Charlson Comorbidity Index (CCI) were used to measure comorbidities. Cerebrovascular disease was classified as present or absent. The CCI comprises a sum of 19 disease categories with assigned values [15]. We divided the CCI into three comorbidity grades (0, 1, and ≥2), with higher scores representing higher comorbidity. Basic activities of daily living (ADL) were measured using the Barthel Index (BI) [16]. These scores ranged 0–100, with higher scores representing greater independence. According to the BI, we divided the physical function of respondents into two categories: >60 (higher independence for basic ADL) and ≤60 (lower independence for basic ADL). Nutritional status was assessed using the Mini Nutritional Assessment Short Form (MNA-SF) [17]. The score ranged 0–14, with higher scores representing better nourishment. According to the MNA-SF, nutritional status was divided into three categories: malnutrition (0–7 points), at risk of malnutrition (8–11 points), and well nourished (12–14 points).

### Statistical analysis

We followed the STROBE guidelines for the analysis of observational data [18]. Descriptive statistics were used to analyze respondents. Linear regression models were used to assess univariate and multivariate associations between total-FTUs and MMSE scores. Multivariate models included sex, age, and all other independent variables significant at *P* < .20 in the univariate regression analysis. When collinearity was a problem, *a priori* regarding inclusion of covariates in the multivariable model was decided. Multicollinearity was assessed using a variance inflation factor cutoff of 4.0. Therefore, denture use demonstrating multicollinearity with total-FTUs was not included in the multivariate model. The models were initially adjusted for age, sex, and natural teeth number (Model 1), then additionally for the longest job and drinking history (Model 2), then additionally for CCI and BI (Model 3), and then finally additionally for MNA-SF (Model 4). Comparison of these models allowed exploration of possible relationship mechanisms between total-FTUs and MMSE scores. All analyses were performed using SPSS statistical software version 21 (SPSS Inc., Chicago, IL, USA), with a significance level of 5%.

## Results

The characteristics of all respondents (N = 200; average age = 85.5 ± 7.8 years for men and 89.4 ± 6.5 years for women) by age categories are presented in Table 1. Of the respondents, 25.0% belonged to the ≤84 years age group, 24.5% to the 85–89 years age group, 30.0% to the 90–94 years age group, and 20.5% to the ≥95 years age group. The mean number of natural teeth was 4.9 ± 7.7 and 54.5% of the respondents had no teeth, and 73.0% used dentures. The mean total-FTUs and MMSE scores was 9.3 ± 4.6 and 11.0 ± 8.6, respectively.

**Table 1:** Characteristics of the Study Population by Age Category.

	All (n = 200)	≤84 years (n = 50)	85–89 years (n = 49)	90–94 years (n = 60)	≥95 years (n = 41)
Sex, n (%)					
Male	45 (22.5)	17 (34.0)	14 (28.6)	9 (15.0)	5 (12.2)
Female	155 (77.5)	33 (66.0)	35 (71.4)	51 (85.0)	36 (87.8)
Longest job, n (%)					
White-collar worker	48 (24.0)	15 (30.0)	12 (24.5)	14 (23.3)	7 (17.1)
Blue-collar worker	108 (54.0)	22 (44.0)	30 (61.2)	30 (50.0)	26 (63.4)
The others including the unemployed	44 (22.0)	13 (26.0)	7 (14.3)	16 (26.7)	8 (19.5)
Smoking history, n (%)					
Never smoker	161 (80.5)	38 (76.0)	35 (71.4)	51 (85.0)	37 (90.2)
Ever or current smoker	39 (19.5)	12 (24.0)	14 (28.6)	9 (15.0)	4 (9.8)
Drinking history, n (%)					
Never drinker	176 (88.0)	41 (82.0)	39 (79.6)	59 (98.3)	37 (90.2)
Ever or current drinker	24 (12.0)	9 (18.0)	10 (20.4)	1 (1.7)	4 (9.8)
Cerebrovascular disease, n (%)					
Yes	82 (41.0)	22 (44.0)	25 (51.0)	21 (35.0)	14 (34.1)
No	118 (59.0)	28 (56.0)	24 (49.0)	39 (65.0)	27 (65.9)
Charlson comorbidity index, n (%)					
0	15 (7.5)	5 (10.0)	2 (4.1)	4 (6.7)	4 (9.8)
1	44 (22.0)	7 (14.0)	12 (24.5)	17 (28.3)	8 (19.5)
≥2	141 (70.5)	38 (76.0)	35 (71.4)	39 (65.0)	29 (70.7)
Barthel Index, n (%)					
≤60	168 (84.0)	42 (84.0)	39 (79.6)	50 (83.3)	37 (90.2)
>60	32 (16.0)	8 (16.0)	10 (20.4)	10 (16.7)	4 (9.8)
Mini Nutritional Assessment Short Form, n (%)					
Malnutrition	33 (16.5)	8 (16.0)	7 (14.3)	12 (20.0)	6 (14.6)
At risk of malnutrition	139 (69.5)	32 (64.0)	36 (73.5)	41 (68.3)	30 (73.2)
Well nourished	28 (14.0)	10 (20.0)	6 (12.2)	7 (11.7)	5 (12.2)
Number of natural teeth, n (%)					
0 teeth	109 (54.5)	26 (52.0)	22 (44.9)	35 (58.3)	26 (63.4)
1–9 teeth	50 (25.0)	11 (22.0)	14 (28.6)	16 (26.7)	9 (22.0)
10–19 teeth	21 (10.5)	5 (10.0)	6 (12.2)	6 (10.0)	4 (9.8)
≥20 teeth	20 (10.0)	8 (16.0)	7 (14.3)	3 (5.0)	2 (4.9)
Denture use, n (%)					
Yes	146 (73.0)	33 (66.0)	37 (75.5)	41 (68.3)	35 (85.4)
No	54 (27.0)	17 (34.0)	12 (24.5)	19 (31.7)	6 (14.6)
Total number of functional tooth units, mean ± SD	9.3 ± 4.6	8.7 ± 5.2	10.0 ± 4.0	8.6 ± 5.0	10.2 ± 3.9
Mini-Mental State Examination score, mean ± SD	11.0 ± 8.6	10.8 ± 9.3	12.4 ± 8.5	10.6 ± 8.9	10.3 ± 7.4

SD = standard deviation.

Table 2 shows the associations of MMSE scores with total-FTUs and covariates, as determined using unadjusted linear regression. These findings showed that total-FTUs significantly positively associated with MMSE scores. Moreover, the longest job, drinking history, CCI, BI, MNA-SF, natural teeth number, and denture use that were significant at *P* < .20 were associated with MMSE scores, but denture use was not included in multivariate adjusted linear regression models because of multicollinearity with total-FTUs.

**Table 2:** Unadjusted Linear Regression Model Coefficients for Mini-Mental State Examination Scores with Total Number of Functional Tooth Units and Covariates.

Characteristic	B (95% CI)	*P*-Value
Age (reference ≤84 years)		
85–89 years	1.59 (-1.83–5.01)	.36
90–94 years	-0.20 (-3.46–3.06)	.91
≥95 years	-0.44 (-4.02–3.15)	.81
Male	1.84 (-1.03–4.71)	.21
Longest job (reference blue-collar worker)		
White-collar worker	3.69 (0.77–6.60)	.01
The others including the unemployed	1.08 (-1.92–4.08)	.48
Never smoker	1.27 (-1.76–4.30)	.41
Never drinker	-4.10 (-7.75–0.44)	.03
Cerebrovascular disease	-0.08 (-2.52–2.37)	.95
Charlson comorbidity index (reference ≥2)		
0	8.12 (3.63–12.61)	< .001
1	0.29 (-2.56–3.14)	.84
Barthel Index >60	8.05 (4.97–11.13)	< .001
Mini Nutritional Assessment Short Form (reference at risk of malnutrition)		
Malnutrition	-6.07 (-9.15–2.99)	< .001
Well nourished	5.08 (1.79–8.38)	.003
Number of natural teeth (reference 0 teeth)		
1–9 teeth	-2.35 (-5.23–0.54)	.11
10–19 teeth	1.86 (-2.17–5.89)	.36
≥20 teeth	-1.57 (-5.68–2.54)	.45
Denture use	4.49 (1.86–7.13)	.001
Total number of functional tooth units	0.47 (0.22–0.72)	< .001

CI = confidence interval.

Table 3 shows the results of the sequence of multivariate analyses. After adjusting for age, sex, and the number of natural teeth (Model 1), total-FTUs was significantly positively associated with MMSE scores. In Model 1, for every 1-point increase in total-FTUs, MMSE scores increased by 0.48 points (*P* < .001). This association remained significant but was attenuated after adjustment for covariates, particularly when comorbidities, basic ADL, and nutritional status were accounted for. After adjusting for all covariates (Model 4), a 1-point increase in total-FTUs was significantly associated with a 0.25-point increase in MMSE scores (*P* = .04). Model 4 also shows that MMSE scores of white-collar workers were 4.29 points higher than those of blue-collar workers (*P* = .001). In respondents with CCI grade 0, MMSE scores were 6.05 points higher than those of respondents with CCI grade >2 (*P* = .005). In respondents with higher independence for basic ADLs, MMSE scores were 4.74 points higher than those of respondents with lower independence for basic ADLs (*P* = .004). Moreover, in respondents with malnutrition, MMSE scores were 4.49 points lesser than those of respondents at risk of malnutrition (*P* = .004). Exploring further the role of nutritional status as a covariate, interaction terms between total-FTUs and MNA-SF were not significant (*P* = .89) (data not shown).

**Table 3:** Multivariable Adjusted Linear Regression Model Coefficients for Mini-Mental State Examination Scores with Total Number of Functional Tooth Units and Covariates.

	Model 1		Model 2		Model 3		Model 4	
Characteristic	B (95% CI)	*P*-Value	B (95% CI)	*P*-Value	B (95% CI)	*P*-Value	B (95% CI)	*P*-Value
Age (reference ≤84 years)								
85–89 years	1.04 (-2.30–4.38)	.54	1.31 (-1.97–4.59)	.43	1.46 (-1.62–4.54)	.35	1.86 (-1.16–4.88)	.23
90–94 years	-0.11 (-3.34–3.11)	.94	0.61 (-2.58–3.79)	.71	0.88 (-2.12–3.87)	.56	1.21 (-1.73–4.15)	.42
≥95 years	-1.17 (-4.75–2.41)	.52	-0.39 (-3.93–3.14)	.83	0.18 (-3.11–3.47)	.91	0.40 (-2.82–3.61)	.81
Male	0.74 (-2.15–3.63)	.62	-0.04 (-3.13–3.06)	.98	0.73 (-2.18–3.64)	.62	0.84 (-2.00–3.67)	.56
Longest job (reference blue-collar worker)								
White-collar worker			4.47 (1.64–7.31)	.002	4.11 (1.46–6.75)	.003	4.29 (1.71–6.88)	.001
The others including the unemployed			1.28 (-1.67–4.22)	.39	0.25 (-2.55–3.05)	.86	0.80 (-1.95–3.55)	.57
Never drinker			-3.69 (-7.70–0.31)	.07	-4.00 (-7.76–0.24)	.04	-3.67 (-7.36–0.03)	.05
Charlson comorbidity index (reference ≥2)								
0					6.39 (2.10–10.67)	.004	6.05 (1.87–10.23)	.005
1					-0.41 (-3.11–2.30)	.77	-0.55 (-3.19–2.10)	.68
Barthel Index >60					6.45 (3.43–9.46)	< .001	4.74 (1.51–7.96)	.004
Mini Nutritional Assessment Short Form (reference at risk of malnutrition)								
Malnutrition							-4.49 (-7.49–1.49)	.004
Well nourished							2.59 (-0.74–5.93)	.13
Number of natural teeth (reference 0 teeth)								
1–9 teeth	-2.06 (-4.90–0.77)	.15	-2.25 (-5.07–0.56)	.12	-1.84 (-4.52–0.84)	.18	-1.64 (-4.26–0.98)	.22
10–19 teeth	2.73 (-1.23–6.70)	.18	3.25 (-0.64–7.13)	.10	2.58 (-1.09–6.25)	.17	3.00 (-0.59–6.59)	.10
≥20 teeth	-1.50 (-5.58–2.57)	.47	-1.80 (-5.79–2.18)	.37	-2.84 (-6.58–0.90)	.14	-3.27 (-6.93–0.39)	.08
Total number of functional tooth units	0.48 (0.22–0.74)	< .001	0.47 (0.21–0.72)	< .001	0.36 (0.11–0.60)	.004	0.25 (0.01–0.49)	.04

CI = confidence interval. Denture use not included in the multivariate model because of multicollinearity.

## Discussion

To the best of our knowledge, this is the first study to comprehensively examine the association between posterior occlusion with natural or artificial teeth and cognitive function among older adults by adjustment for the effects of natural teeth number and other possible confounders. This study showed that Japanese nursing home residents with loss of posterior teeth occlusion had significantly lower cognitive function. When posterior teeth occlusion and number of natural teeth were entered simultaneously into the regression analysis, this tendency remained significant, but there was non-significant association between the number of natural teeth and cognitive function. Moreover, this tendency was observed even after adjustment for all other covariates.

Although prior studies indicate that tooth loss can be considered an early marker of cognitive decline in older adults [5–10], our study demonstrates that loss of functional posterior tooth units may be more closely associated with cognitive decline than tooth loss. Our previous report supports the present findings. In a Japanese community-dwelling older population, loss of functional teeth appeared related with increased likelihood of lower cognitive function levels [19]. However, one community-based cross-sectional study did not show a significant association between occluding pattern of posterior teeth and cognitive function [11]. Although this previous study roughly classified posterior teeth occlusion (total, partial, or lost support) in community-dwelling older adults, the present study treated posterior teeth occlusion as continuous values within a fixed range in nursing home older adults. Thus, the differences in the findings of previous study might be owing to variations in measurements and sample characteristics.

We also confirmed significant associations of cognitive function with socioeconomic status (as indicated by longest job), medical comorbidity, basic ADLs, and nutritional status. Our results supported previous findings that lower occupation-based socioeconomic status was significantly associated with lower MMSE scores and increased risks of Alzheimer’s disease [20, 21]. In addition, our findings are generally consistent with those of previous studies indicating the significant positive associations of MMSE scores with CCI, BI, and MNA [22, 23].

There are two plausible relationship mechanisms between loss of posterior teeth occlusion and cognitive decline among older adults. One may be a nutritional pathway. Some epidemiological studies indicate that dental and oral health problems, such as tooth loss and dysphagia, are associated with nutritional status [24–27]. In particular, impaired posterior teeth occlusion measured using total-FTUs has been suggested to contribute to poor nutritional status [28]. In addition, subsequent poor nutrition-associated problems, such as lack of antioxidant nutrients and dietary lipids, result in risk factors for cognitive decline [29, 30]. Furthermore, the present study confirmed that the association of cognitive function with posterior teeth occlusion was considerably attenuated when nutritional status was controlled in analytical models and nutritional status itself was associated with cognitive function in the fully adjusted model. This highlighted the importance role of nutritional status in explaining the association between posterior teeth occlusion and cognitive function. Another suggested pathway is that the increase in cerebral blood flow, activation of cortical area, and blood oxygen levels resulting from masticatory stimulation with normal occlusion activate brain function [31–34]. Thus, reduced mastication resulting from loss of posterior teeth occlusion might cause cognitive decline. This was supported by previous studies, which indicated that poorer masticatory performance (as indicated by measurements such as color-changeable gum, self-reported questionnaire, and mastication score of food items) lowers cognitive function among older adults [35–38]. In addition, our results may support the earlier-described pathway. There was a significant direct positive association with cognitive function for posterior teeth occlusion even after adjustment for all covariates as possible confounders. However, we could not ascertain variations in brain activation by mastication through our data set. In addition, we could not rule out the possibility that reduced mastication may have caused the hippocampal functional morphological changes, which have been reported to induce spatial memory and learning deficits [39].

The present findings have public health implications. Early detection of contributing factors to cognitive decline is a priority in healthcare systems. A systematic literature review suggests that 24.3 million people suffer from dementia worldwide, with 4.6 million new cases of dementia every year [40]. In the general Japanese population, which has a very high proportion of older individuals, the reported prevalence of all-cause dementia has increased over the past 20 years [41]. In our analyses, total-FTUs and MMSE scores were used in continuous formats to estimate the association between posterior teeth occlusion and cognitive function. The resulting linear association was evident, suggesting that gradual decrease in total-FTUs might affect the individual’s cognitive function with any score of total-FTUs. Thus, we suggest that loss of posterior teeth occlusion as indicated by total-FTUs is possibly a modifiable risk factor for cognitive decline in our aging society. Loss of posterior teeth occlusion can be restored by providing artificial teeth on fixed and/or removable prostheses, even if individuals have multiple tooth loss. Therefore, promoting and supporting opportunities for dental care and treatment, especially for maintenance and restoration of posterior teeth occlusion, as a public health intervention may contribute to the prevention of cognitive decline in aged populations.

Our study has several limitations. First, this was cross-sectional study. Therefore, it was impossible to generate any statements on causation or to exclude the possibility of reverse causation, in that, decline in cognitive function affects posterior teeth occlusion. For example, cognitive decline may be present with physical decline and may be a barrier to access to dental care, which makes it difficult to maintain and restore posterior teeth occlusion. Accordingly, longitudinal or interventional studies are needed to determine the effects of loss of posterior teeth occlusion on cognitive function. Second, the outcome measure was only based on a cognitive screening assessment and therefore, could not cover all domains of cognitive function. Third, lack of information regarding potential confounding factors related to cognitive decline, such as frailty [42], depression [43], and psychotropic medication use [44], may have reduced the accuracy of our findings to some extent. Therefore, the influence of these unadjusted confounding factors should be evaluated in future. Lastly, the data we used for our analysis were based on a relatively small sample taken from nursing home older residents in one medium-sized municipality in Japan. Caution should be exercised when interpreting our results, because our findings may not be generalizable across all nursing home settings or all older adults.

In conclusion, the present study demonstrated that loss of posterior teeth occlusion was independently associated with cognitive decline in nursing home older residents in Japan. Our findings highlight maintenance and restoration of posterior teeth occlusion as a possible preventive factor against cognitive decline in aged populations.
